# FGF21 as an Endocrine Regulator in Lipid Metabolism: From Molecular Evolution to Physiology and Pathophysiology

**DOI:** 10.1155/2011/981315

**Published:** 2011-02-06

**Authors:** Yusuke Murata, Morichika Konishi, Nobuyuki Itoh

**Affiliations:** Department of Genetic Biochemistry, Kyoto University Graduate School of Pharmaceutical Sciences, Sakyo, Kyoto 606-8501, Japan

## Abstract

The FGF family comprises twenty-two structurally related proteins with functions in development and metabolism. The *Fgf21* gene was generated early in vertebrate evolution. FGF21 acts as an endocrine regulator in lipid metabolism. Hepatic *Fgf21* expression is markedly induced in mice by fasting or a ketogenic diet. Experiments with *Fgf21* transgenic mice and cultured cells indicate that FGF21 exerts pharmacological effects on glucose and lipid metabolism in hepatocytes and adipocytes via cell surface FGF receptors. However, experiments with *Fgf21* knockout mice indicate that FGF21 inhibits lipolysis in adipocytes during fasting and attenuates torpor induced by a ketogenic diet but maybe not a physiological regulator for these hepatic functions. These findings suggest the pharmacological effects to be distinct from the physiological roles. Serum FGF21 levels are increased in patients with metabolic diseases having insulin resistance, indicating that FGF21 is a metabolic regulator and a biomarker for these diseases.

## 1. Background

Prototypes of fibroblast growth factors (FGFs), FGF1 and FGF2, were originally isolated as mitogens for cultured fibroblasts from the brain and pituitary [1, 2]. The human/mouse *Fgf* gene family comprises twenty-two members, including *Fgf1*–*Fgf23*, all of which are evolutionarily related. However, as mouse *Fgf15* and human *Fgf19* are orthologous, we refer to these genes as *Fgf15/19 *in this paper. Human/mouse FGFs are proteins of ~150–300 amino acids with 13–71% identity. All FGFs with a ~120 amino acid conserved core region (~30–60% identity) are signaling molecules with diverse functions in development and metabolism. *Fgf *genes are widely expressed in developing and adult tissues [3–6].

FGFs can be classified into three types, paracrine, intracrine, and endocrine FGFs, by their mechanisms of action [6]. Paracrine FGFs (FGF1~FGF10, FGF16~FGF18, FGF20, and FGF22) function as secreted local paracrine signaling molecules in multiple developmental processes, including differentiation, cell proliferation, and migration. They mediate biological responses by binding to cell surface tyrosine kinase FGF receptors (FGFRs) [4, 6, 7]. Intracrine FGFs (FGF11~FGF14) function as nonsecreted signaling molecules. They mainly play roles in neuronal functions at postnatal stages in an FGFR-independent manner [8–10]. Endocrine FGFs (FGF15/19, FGF21, and FGF23) mediate biological responses as secreted proteins in an FGFR-dependent manner. Endocrine FGFs function over long distances in an endocrine manner and mainly play roles in metabolism at postnatal stages [11–13].

Paracrine and intracrine FGFs have been identified in both invertebrates and vertebrates. However, endocrine FGFs have only been identified in vertebrates [6]. Endocrine FGFs are emerging in evolution. FGF15/19 and FGF23 play roles as metabolic regulators in bile acid metabolism and phosphate and active vitamin D metabolism, respectively [11, 13–15]. FGF21 exerts diverse pharmacological effects on glucose and lipid metabolism, ketogenesis, and growth hormone signaling in hepatocytes in mice. However, FGF21 may not be a physiological regulator for these hepatic functions. FGF21 physiologically regulates lipid metabolism in adipocytes and torpor. Serum FGF21 levels are significantly increased in patients with metabolic diseases having insulin resistance. These results indicate the physiological and pathophysiological roles of FGF21. Several excellent review articles on FGF21, focusing on its pharmacological effects on metabolism and therapeutic uses for metabolic diseases have been published [16–20]. This paper concentrates on the molecular evolution and physiological and pathophysiological roles of FGF21.

## 2. Identification of *Fgf21*

The *Fgf21* gene was originally identified in mice by the polymerase chain reaction with the amino acid sequence of human FGF15/19. Human *Fgf21* was also identified by homology-based searching in the human DNA database [21]. Later, human FGF21 was found to act as a stimulator of glucose uptake in mouse 3T3-L1 adipocytes in an assay used to search for novel proteins with therapeutic potential to treat diabetes [22]. Mouse *Fgf21* was also identified as a hepatic gene inducible by fasting or a ketogenic diet [23, 24].

Human FGF21 is a secreted protein of 209 amino acids with a 29-amino-acid amino-terminal secreted signal sequence and a ~120-amino-acid conserved core region. Human FGF21 is highly homologous to mouse FGF21 (~75% identity). However, its low homology with other human FGFs (less than 35% identity) indicates that FGF21 is structurally unique [21]. Paracrine FGFs have a heparin-binding site, which is necessary for stable interaction with FGFRs and heparin-like molecules [25]. However, FGF21 as well as FGF15/19 and FGF23 lack a typical heparin-binding site [25, 26].

## 3. Molecular Evolution of *Fgf21*

**Figure 1:**
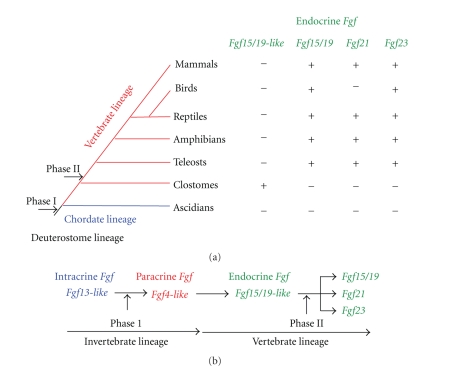
The evolutionary lineage of deuterostome organisms and the evolutionary history of endocrine* Fgf* genes. (a) The *Fgf* gene family expanded in two major phases (I and II) during deuterostome evolution. Phase I occurred after the separation of protostomes and deuterostomes. Phase II occurred early in the emergence of vertebrates. (b) *Fgf13-like *is the ancestral gene of the *Fgf* gene family. *Fgf4-like* was generated from *Fgf13-like* by gene duplication during invertebrate evolution. *Fgf15/19-like *was also generated from *Fgf4-like* by local gene duplication early in the emergence of vertebrates. Later, Fgf19, Fgf21, and Fgf23 were generated via two genome duplication events in phase II.

The FGF signaling system has been conserved throughout metazoan evolution. Potential evolutionary relationships in the *Fgf* family have been proposed based on results of gene location and phylogenetic analyses. These analyses have identified seven subfamilies: *Fgf/1/2/5*, *Fgf3/4/6*, *Fgf7/10/22*, *Fgf8/17/18*, *Fgf9/16/20*, *Fgf11/12/13/14*, and *Fgf15/19/21/23* [4, 6]. Ascidians belong to the subphylum Urochordata, the earliest branch in the phylum Chordata. Ancestral genes of paracrine and intracrine *Fgf*s have been identified in the ascidian, *Ciona intestinalis* [27]. However, no ancestral gene of endocrine* Fgfs* has been identified in *Ciona intestinalis*. The sea lampreys, *Petromyzon marinus*, are cyclostomes, the most basal extant group of vertebrates [28]. An ancestral gene of endocrine* Fgf*s has been identified in the lamprey genome, and tentatively named *Fgf15/19-like* (Itoh et al., unpublished observation) (Figure 1(a)). Lamprey FGF15/19-like also lacks a typical heparin-binding site. These results support that lamprey *Fgf15/19-like* is an ancestral endocrine *Fgf*, which was generated from the ancestral paracrine *Fgf *gene, *Fgf4-like*, by local gene duplication early in vertebrate evolution (Figure 1(b)). Later, *Fgf19*, *Fgf21*, and *Fgf23* were generated from the ancestral endocrine *Fgf *gene by two genome duplication events early in vertebrate evolution [4, 6]. The evolutionary history suggests that endocrine FGFs are vertebrate specific. As described above, paracrine FGFs have a heparin-binding site. The site is necessary for the stable binding of FGFRs/heparin-like molecules and local signaling in a paracrine manner. Endocrine FGFs potentially acquired systemic signaling in an endocrine manner by reducing heparin-binding affinity early in vertebrate evolution [6, 13, 25].

Endocrine *Fgf *genes have been identified in all vertebrates examined, including teleosts, amphibians, reptiles, birds, and mammals. *Fgf21* has also been identified in most vertebrates. However, *Fgf21* has not been identified in the chicken and zebra finch genomes (Ensemble Genome Browser; Itoh et al., unpublished observation) (Figure 1(a)). Genome sizes and gene numbers are smaller in birds than in mammalian species. Although the evolutionary implications of these changes remain to be understood, the reduced genome sizes and gene numbers may have evolved in response to the physiological demands of flight [29]. *Fgf21* might therefore have been lost in the bird lineage.

## 4. Roles of FGF21 in Glucose Metabolism

*Fgf21* is expressed abundantly in the liver, and also in the pancreas, white adipose tissue, muscle, and testis [17, 21, 30]. Potential roles of FGF21 in glucose metabolism were first shown by experiments with cultured cells [22]. FGF21 stimulated glucose uptake in cultured mouse and human adipocytes. Functional interplay between the FGF21 and peroxisome proliferation-activated receptor *γ* (PPAR*γ*) pathways led to a marked stimulation of glucose transport, suggesting a novel synergy between FGF21 and PPAR*γ* homeostasis [31]. In addition, *Fgf21* transgenic mice were resistant to diet-induced obesity. Serum glucose levels were also reduced to near normal levels in both *ob/ob* and *db/db* mice by the administration of FGF21. These findings indicate that FGF21 plays a role in glucose metabolism and has potential therapeutic effects on metabolic diseases [16–19].

To elucidate the physiological roles of FGF21, *Fgf21* knockout mice have been generated. These mice had normal food intake and energy expenditure levels, serum glucose and insulin levels, and hepatic glycogen levels, indicating that FGF21 is not to be a physiological regulator for glucose metabolism [32].

## 5. Roles of FGF21 in Lipolysis in Adipocytes

**Figure 2:**
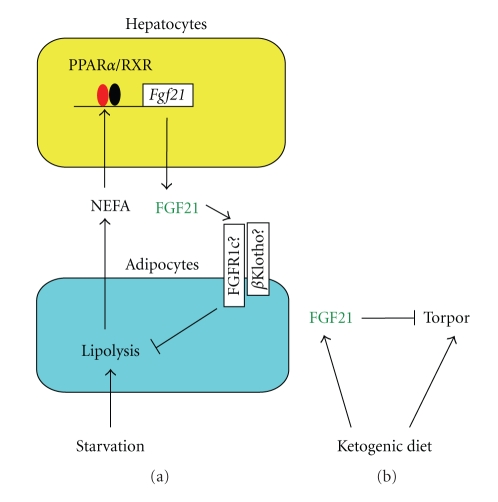
Mechanism of action and regulatory mechanism of the gene expression of FGF21. (a) Hepatic *Fgf21* expression is induced by the activation of PPAR*α*. NEFA binds to and activates PPAR*α*. The ligand-bound PPAR*α* forms a heterodimer with RXRs and induces the expression of *Fgf21*. The FGF21 inhibits lipolysis in adipocytes. In cultured adipocytes, FGF21 signaling is transduced by activating the *β*Klotho-FGFR1c complex. However, FGF21 signaling can be transduced in *βKlotho* knockout mice, indicating the existence of a *β*Klotho-independent FGF21 signaling pathway. The regulatory process forms a negative feedback loop in the control of lipolysis by FGF21. (b) Feeding with a ketogenic diet (KD) mimics the metabolic conditions of chronic starvation. KD induces hepatic *Fgf21* expression and torpor. FGF21 attenuates torpor induced by KD.

 Mammals have evolved complex metabolic responses to fasting. During fasting, nonesterified fatty acid (NEFA) is released from adipocytes into the blood and taken up by hepatocytes. Peroxisome proliferator-activated receptor *α* (PPAR*α*) is a nuclear receptor. Hepatic *Fgf21* expression was greatly induced by fasting for 24 h in wild-type mice but not PPAR*α* knockout mice. It was also markedly induced by a PPAR*α*-selective agonist [23]. These results indicate that hepatic *Fgf21* expression is induced by the activation of PPAR*α*. NEFA binds to and activates PPAR*α*. The ligand-bound PPAR*α* forms a heterodimer with RXRs and induces the expression of *Fgf21* [33]. Fasting increases the amount of NEFA released from adipocytes. Hepatic *Fgf21* expression during fasting is probably induced through the activation of PPAR*α* by NEFA (Figure 2(a)) [34, 35]. *Fgf21* knockout mice fasted for 24 h showed increased lipolysis in adipocytes, which resulted in decreased adipocyte size and increased serum NEFA levels [32]. These results indicate that FGF21 inhibits lipolysis in adipocytes during fasting. The regulatory process forms a negative feedback loop in the control of lipolysis by FGF21 (Figure 2(a)). FGF21 also regulates mitochondrial activity and enhances oxidative capacity through an AMP-activated protein kinase- (AMPK-) sirtuin 1- (SIRT1-) peroxisome proliferator-activated receptor-*γ* coactivator-1*α*- (PGC-1*α*-) dependent mechanism in adipocytes [36].

## 6. Roles of FGF21 in Ketogenesis and Triglyceride Clearance in Hepatocytes

In hepatocytes, NEFA is converted to acetyl-CoA by oxidation, and ketone bodies are produced from acetyl-CoA. Ketone bodies become the predominant energy source for the brain during fasting. Hepatic ketogenesis during fasting was greatly impaired in *PPAR*α** knockout mice, indicating that PPAR*α* is crucial to the normal adaptive response to fasting [37, 38]. As described above, hepatic *Fgf21* expression is induced in response to fasting and PPAR*α* agonists. In addition, the phenotypes of *Fgf21* transgenic mice demonstrate that FGF21 stimulates hepatic ketogenesis, indicating that FGF21 plays a role in hepatic ketogenesis [23]. Feeding with a ketogenic diet (KD) mimics the metabolic conditions of chronic starvation. Adenoviral knockdown of hepatic *Fgf21* in mice fed KD caused reduced blood ketone levels, fatty liver, and lipemia, suggesting that FGF21 is required for hepatic ketogenesis and triglyceride clearance in mice fed KD [24]. In addition, serum triglyceride levels were reduced to near normal levels in both *ob/ob* and *db/db* mice by the administration of FGF21 [22]. These findings also indicate functions of FGF21 in ketogenesis and triglyceride metabolism and potential therapeutic effects on metabolic diseases [16–19]. However, hepatic ketogenesis and triglyceride levels were essentially normal in *Fgf21* knockout mice fasted or fed KD, indicating FGF21 not to be a physiological regulator for hepatic ketogenesis and triglyceride clearance in mice [32]. These results suggest the physiological roles of FGF21 to be distinct from the pharmacological effects of FGF21 indicated by experiments with *Fgf21* transgenic mice. In humans, serum FGF21 levels are also increased by fasting for 7 days or PPAR*α* activation. In contrast, ketogenesis is independent of serum FGF21 levels [39, 40]. However, it also has been reported that *Fgf21* knockout mice fed KD developed hepatosteatosis and showed partial impairment in ketogenesis [41].

Peroxisome proliferation-activated receptor *γ* coactivator-1*α* (PGC-1*α*) regulates metabolism in response to changes in nutritional status. PGC-1*α* negatively regulated hepatic F*gf21* expression [42]. In contrast, FGF21 induced hepatic *Pgc-1*α** expression. *Fgf21* knockout mice did not fully express *Pgc-1*α** in response to prolonged fasting and exhibited impaired gluconeogenesis and ketogenesis. In addition, FGF21 could not induce gluconeogenic gene expression in *Pgc-1*α** knockout mice [43]. These results indicate that gluconeogenesis and ketogenesis by FGF21 are mediated in part through PGC-1*α*. However, as described above, other experiments with *Fgf21* knockout mice suggest that FGF21 may not be required for gluconeogenesis and ketogenesis [32].

## 7. Roles of FGF21 in Growth Hormone Signaling in Hepatocytes

Starvation inhibits growth by blocking the growth hormone (GH)/insulin-like growth factor 1 (IGF1) signaling pathway [44]. *Fgf21* transgenic mice are 40–50% smaller than their wild-type counterparts. Tibia length is also significantly reduced in *Fgf21* transgenic mice. FGF21 causes resistance to GH in the liver [45]. Actions of GH are mostly mediated by IGF1. *Igf1* expression is induced by the GH/STAT5 (signal transducer and activator of transcription 5) signaling pathway. The phosphorylation of STAT5 and the expression of *Igf1* are significantly decreased in livers of *Fgf21* transgenic mice. IGF-binding protein 1 (IGFBP1), which is involved in sequestering IGF1, inhibits IGF1 signaling. A suppressor of cytokine signaling 2 (SOCS2) also inhibits GH signaling by binding to the tyrosine-phosphorylated GH receptor. The expression of *Igfbp1* and *Socs2* was greatly enhanced in *Fgf21* transgenic livers. These results indicate the important role of FGF21 in the inhibition of GH/IGF1 signaling [45]. However, *Fgf21* knockout mice are apparently healthy, and their body and tibia lengths are essentially normal [32]. In addition, hepatic *Igf1*, *Igfbp1*, and *Socs2* expression was essentially normal in *Fgf21* knockout mice (Murata et al., unpublished observation). The expression of *Igf1* was slightly decreased by fasting for 24 h in wild-type mice. In contrast, the expression of *Igfbp1* and *Socs2* was greatly and slightly increased in fasted wild-type mice, respectively. In addition, the expression of *Igf1*, *Igfbp1*, and *Socs2* in fasted *Fgf21* knockout mice was similar to that in fasted wild-type mice (Murata et al., unpublished observation). The *Fgf21* knockout phenotypes indicate that FGF21 is not a physiological regulator essential for GH/IGF1 signaling.

## 8. Roles of FGF21 in Torpor

Torpor, the controlled lowering of metabolic rates, body temperature, and physical activity, is an adaptation that various mammals use to cope with periods of low food availability [46]. The basal core body temperature of *Fgf21* transgenic mice is consistently lower than that of wild-type mice. Moreover,* Fgf21* transgenic mice enter torpor on fasting for 24 h, whereas wild-type mice do not [23]. In addition, a PPAR pan-agonist reduced body temperature late at night in concert with the induction of hepatic of *Fgf21* expression [47]. However, body temperature and physical activity were essentially normal in *Fgf2*1 knockout mice fasted for 24 h, indicating that FGF21 is not physiologically required for torpor induced by fasting for 24 h [48]. Hepatic *Fgf21* expression and torpor were also induced in mice fed KD for 5 days (Figure 2(b)). However, torpor was attenuated in *Fgf21* knockout mice fed KD for 5 days. These results indicate that FGF21 is potentially involved in the torpor induced by KD [48] (Figure 2(b)).

## 9. Mechanism of FGF21 Action

FGF signaling is mostly mediated by the activation of FGFRs. Four *Fgfr *genes, *Fgfr1*–*Fgfr4,* have been identified in humans and mice [3, 7, 49]. These genes encode proteins (~800 amino acids) that contain an extracellular ligand-binding domain with three immunoglobulin-like domains (I, II, and III), a transmembrane domain, and intracellular tyrosine kinase domains. *Fgfr1*–*Fgfr3* encode two major variants of immunoglobin-like domain III (IIIb and IIIc) generated by alternative splicing. The immunoglobulin-like domain III is an essential determinant of ligand-binding specificity [26]. Thus, seven FGFR proteins (FGFR 1b, 1c, 2b, 2c, 3b, 3c, and 4) with differing ligand-binding specificity are generated from *Fgfr1*–*Fgfr4*. The binding of FGFs to FGFRs induces receptor dimerization and transphosphorylation and the activation of downstream signaling pathways: RAS-RAF-MAPK, PI3K-AKT, STAT, and PLC*γ* [7, 49].

Endocrine FGFs also mediate biological responses in an FGFR-dependent manner. However, they activate FGFRs with very low activity even in the presence of heparin/heparan sulfate, as they bind to heparin/heparan sulfate with very low affinity [25, 26]. *α*Klotho is a transmembrane protein of ~1,000 amino acids with a short cytoplasmic domain [50]. *β*Klotho shares structural identity (41% amino acid identity) and characteristics with *α*Klotho [51]. *Fgf23* and *αKlotho* knockout mouse phenotypes indicate that FGF23 and *α*Klotho function in a common signal transduction pathway. FGF23 can bind to the *α*Klotho-FGFR1c complex in cultured cells [52], suggesting that *α*Klotho is a cofactor essential for FGF23 signaling. *βKlotho*, *Fgf15/19*, and *Fgfr4* knockout mouse phenotypes also indicate that FGF15/19, FGFR4, and *β*Klotho function in a common signal transduction pathway [53–55]. FGF15/19 can bind to the *β*Klotho-FGFR4 complex in cultured cells. FGF15/19 also activates FGF signaling in hepatocytes that primarily express *Fgfr4* [56].

In the presence of **β**Klotho, FGF21 can bind to and activate FGFR1c, FGFR2c, FGFR3c, or FGFR4, which activates the MAP kinase pathway, in cultured cells, indicating that *β*Klotho is also essential for FGF21 signaling in cultured cells [57–59]. However, *Fgf21* knockout mouse phenotypes [32] are distinct from *βKlotho* knockout mouse phenotypes [51]. In addition, the administration of a recombinant human FGF21 to *βKlotho* knockout mice showed that FGF21 signaling is transduced in the absence of *β*Klotho [60]. These results indicate the existence of a *β*Klotho-independent FGF21 signaling pathway in which undefined cofactors might be involved [60].

## 10. FGF21 Signaling in Metabolic Diseases

Nonalcoholic fatty liver disease (NAFLD) is a hepatic manifestation of metabolic syndrome and ranges from simple fatty liver to nonalcoholic steatohepatitis. Its prevalence has increased dramatically over recent years in developed countries [61]. The pathophysiological hallmark of NAFLD is insulin resistance. NAFLD may increase the risk of type 2 diabetes and atherosclerosis [62]. Serum FGF21 levels are significantly increased in NAFLD (Table 1) [63–65]. Serum FGF21 levels are positively correlated with intrahepatic triglyceride levels [65]. As NAFLD is now recognized as a major public health problem, reliable biomarkers for NEFLD are needed. Serum FGF21 levels might be useful as a biomarker for NEFLD [61].

Type 2 diabetes connected with visceral obesity and insulin resistance has become a global health concern. Serum FGF21 levels are increased in patients with type 2 diabetes, gestational diabetes, and obesity, indicating FGF21 to be a potential new marker in patients with type 2 diabetes (Table 1) [66–71]. Serum FGF21 levels are independently associated with markers of insulin resistance and an adverse lipid profile [66, 70]. The upregulation of serum FGF21 levels might be a compensatory mechanism to improve glucose metabolism when insulin resistance is present. Diet-induced obese mice also have increased serum (endogenous) FGF21 levels and respond poorly to exogenous FGF21, indicating that obesity is an FGF21-resistant state [72]. Impaired glucose tolerance (IGT) is an important category of prediabetes. Serum FGF21 levels are also increased in Chinese subjects with IGT. However, serum FGF21 levels do not correlate with insulin resistance in the subjects [73].

Cushing's syndrome is a hormone disorder caused by high levels of cortisol (hypercortisolism) in the blood. Patients with Cushing's syndrome frequently suffer from visceral obesity, insulin resistance/diabetes, and other abnormalities similarly to patients with metabolic syndrome. Serum FGF21 levels are also increased in patients with Cushing's syndrome. The increased FGF21 levels are due to excessive fat accumulation and related metabolic abnormalities rather than a direct effect of cortical on FGF21 production (Table 1) [74].

Lipodystrophy is a common alteration in HIV-1-infected patients under antiretroviral treatment. This syndrome is usually associated with peripheral lipoatrophy, central adiposity, and, in some cases, lipomatosis, as well as systemic insulin resistance and hyperlipidemia [75]. Serum FGF21 levels are increased in HIV-1-infected patients with lipodystrophy. This increase is closely associated with insulin resistance, metabolic syndrome, and markers of liver damage. FGF21 might be a biomarker of altered metabolism in HIV-1-infected, antiretroviral-treated patients (Table 1) [76].

Serum FGF21 levels correlate with renal function and are markedly increased in chronic kidney disease patients receiving hemodialysis, suggesting a possible link between their FGF21 levels and renal function [77]. Patients with end-stage renal disease (ESRD) show insulin resistance. Serum FGF21 levels are also markedly increased in patients with ESRD, suggesting FGF21 to play a role in insulin resistance in these patients (Table 1) [78].

## 11. Conclusion

Endocrine FGFs, FGF15/19, FGF21, and FGF23, are emerging in evolution and unique in function. The *Fgf21* gene, which was generated early in vertebrate evolution, is specific to vertebrates. *Fgf21* has been identified in most vertebrate genomes, but not in bird genomes, indicating that it might be lost in the bird lineage. Genome sizes and gene numbers are smaller in birds than in mammalian species. As the differences might have evolved in response to the physiological demands of flight, *Fgf21* also might have been lost. FGF21 mainly acts as an endocrine factor in an FGFR-dependent manner. FGF21 requires *β*Klotho as a cofactor in cultured cells. However, it may not require *β*Klotho in mice. FGF21 exerts pharmacological effects on hepatic glucose and lipid metabolism and growth hormone signaling. These effects might be useful for treating metabolic diseases. However, experiments with *Fgf21* knockout mice indicated FGF21 not to be physiologically essential for hepatic glucose and lipid metabolism, ketogenesis, and growth hormone signaling. In contrast, FGF21 inhibited lipolysis in adipocytes of fasted mice and attenuated torpor induced by KD, indicating that *Fgf21* may be a “thrifty gene.” Serum FGF21 levels are increased in patients with metabolic diseases having insulin resistance including NAFLD, type 2 diabetes, Cushing's syndrome, and HIV-1-induced lipodystrophy. Although it remains unclear whether serum FGF21 levels are increased by FGF21 resistance or an adaptive response to metabolic disorders, these findings indicate that FGF21 potentially functions as a metabolic regulator in relation with insulin resistance and is a biomarker for metabolic diseases. Further study of FGF21 may provide clues as to its roles in lipid metabolism and clinical treatments for metabolic diseases.

## Figures and Tables

**Table 1:** Increased serum FGF21 levels in metabolic disease patients with insulin resistance.

Disease	Insulin signaling	Serum FGF21 levels
Nonalcoholic fatty liver disease	Resistance	Increase
Type 2 diabetes	Resistance	Increase
Obesity	Resistance	Increase
Cushing's syndrome	Resistance	Increase
Lipodystrophy induced by HIV-1	Resistance	Increase
Endostage renal disease	Resistance	Increase
